# Executive function and prospective falls: a 6-year longitudinal study in community-dwelling older adults

**DOI:** 10.1186/s12877-023-03790-9

**Published:** 2023-03-10

**Authors:** Cindi Smith, Laurence Seematter-Bagnoud, Brigitte Santos-Eggimann, Helene Krief, Christophe J. Bula

**Affiliations:** 1 Service of Geriatric Medicine and Geriatric Rehabilitation, Lausanne University Hospital (CHUV), Mont Paisible 16, 1011 Lausanne, Switzerland; 2 Center for Primary Care and Public Health (Unisanté), University of Lausanne, Route de La Corniche 10, 1010 Lausanne, Switzerland

**Keywords:** Falls, Executive functions, Cognitive impairment, Multiple fallers

## Abstract

**Background:**

Older people with impaired executive function (EF) might have an increased fall risk, but prospective studies with prolonged follow-up are scarce. This study aimed to investigate the association between a) EF at baseline; b) 6-year decline in EF performance; and fall status 6 years later.

**Methods:**

Participants were 906 community-dwelling adults aged 65–69 years, enrolled in the Lausanne 65 + cohort. EF was measured at baseline and at 6 years using clock drawing test (CDT), verbal fluency (VF), Trail Making Test (TMT) A and B, and TMT ratio (TMT-B – TMT-A/TMT-A). EF decline was defined as clinically meaningful poorer performance at 6 years. Falls data were collected at 6 years using monthly calendars over 12 months.

**Results:**

Over 12-month follow-up, 13.0% of participants reported a single benign fall, and 20.2% serious (i.e., multiple and/or injurious) falls. In multivariable analysis, participants with worse TMT-B performance (adjusted Relative Risk Ratio, adjRRR_TMT-B worst quintile_ = 0.38, 95%CI:0.19–0.75, *p* = .006) and worse TMT ratio (adjRRR_TMT ratio worst quintile_ = 0.31, 95%CI:0.15–0.64, *p* = .001) were less likely to report a benign fall, whereas no significant association was observed with serious falls. In a subgroup analysis among fallers, participants with worse TMT-B (OR:1.86, 95%CI = 0.98–3.53, *p* = .059) and worse TMT ratio (OR:1.84,95%CI = 0.98–3.43,*p* = .057) tended to have higher odds of serious falls. EF decline was not associated to higher odds of falls.

**Conclusions:**

Participants with worse EF were less likely to report a single benign fall at follow-up, while fallers with worse EF tended to report multiple and/or injurious falls more frequently. Future studies should investigate the role of slight EF impairment in provoking serious falls in active young-old adults.

**Supplementary Information:**

The online version contains supplementary material available at 10.1186/s12877-023-03790-9.

## Introduction

A focus in falls research is the role of cognition in postural control, a complex process involving the coordination of sensory and motor systems through higher-order neurological processes, particularly executive functions (EF) [1, 2]. EF are required for planning movements, dividing attention, and responding to changes in the environment. A recent systematic review identified EF as the cognitive function most associated with fall risk in older people [3]. Another systematic review found that EF was associated with falls and gait speed slowing in older adults [4]. Impairment in cognitive abilities, especially in EF, has been associated with an increased fall risk, even in older individuals without cognitive impairment [5–9]. For example, a study in community-dwelling older adults identified lower performance in verbal fluency as a predictor of recurrent falls over the next 12 months [9]. However, only very few studies examined the dynamic association between EF and fall risk over a prolonged follow-up [3, 7, 9, 10]. One work showed that decline in verbal ability, processing speed, and immediate memory were associated with increased rates of falling and fall risk over an eight-year follow-up period [10]. In contrast, the one study that reported a significant association between EF and falls over a 5-year follow-up period used a single measure of EF at baseline, but did not investigate the association between changes in EF over time and falls in the subsequent years [7]. Furthermore, adjustment for confounders in this study was limited to a few variables that did not reflect the range of potential risk factors for falls.

Studies that showed an association between EF and fall risk have also been criticized because of the heterogeneity of tests used to assess EF performance, ranging from a single test such as the trail making test (TMT) [11], to complex, computerized test battery [5]. Time limitation and the need for specific computer programs or specialized neuropsychological expertise to administer more complex EF tests preclude their use in primary care. Several cross-sectional studies that used simpler tests such as the Clock Drawing Test (CDT) or the Trail Making Test (TMT) showed independent association between impaired performance and fall risk, as well as with fall-related injury [12, 13]. However, the performance of these easier-to-administer tests of EF in predicting fall risk prospectively over a longer range has not been well studied so far.

Finally, another limitation of previous studies relates to the analytic strategy that combined all participants with falls in a single group. Current stratification of fall risk however considers multiple falls and/or injurious falls as indicating a high risk for recurrent falls, whereas single falls indicate intermediate risk [14]. Furthermore, previous research suggests that recurrent falls differ from single falls in several ways including cognition and other risk factors, predictability, as well as response to preventive interventions [15–19]. Similarly, falls with serious injuries have been shown as highly associated with poor global cognitive and EF performance [20, 21].

This study aimed to address several limitations of previous studies in using a prospective design, assessing EF with easy-to-administer tests at two points in time 6 years apart, and collecting prospectively data on subsequent falls.

Specifically, the aims of this study were to investigate the prospective association between EF performance (at baseline and decline over a 6-year period) and fall status 6 years later in young older adults, aged 65 to 69 years at baseline.

Our hypothesis was that participants with worse EF performance at baseline would be more likely to report falls 6 years later, especially multiple and/or injurious falls. Similarly, we hypothesized that, compared to participants with stable EF performance over the 6-year study period, those with decline in EF performance would be more likely to report falls 6 years later, including multiple and/or injurious falls.

## Methods

### Study population

Data for the study were drawn from the Lausanne Cohort 65 + study (Lc65 +) [22, 23]. This cohort enrolled three representative samples of about 1500 community-dwelling residents of the city of Lausanne aged 65 to 69 years in 2004, 2009, and 2014, respectively. For the current study, only data from the first sample were used. Follow-up includes self-completed yearly questionnaires, as well as in-person visits at 3-year intervals from 2005 on (i.e. 2008, 2011) with cognitive and physical performance tests conducted by trained research assistants.

From the 1422 participants to the 2005 baseline assessment, 1006 (70.7%) participated in 2011. Fall status from monthly calendars could be assessed in 906 (90.1%) participants who were included in the analyses (Fig. 1 in Supplementary material).

The Lausanne Cohort 65 + study received approval from the Cantonal Human Research Ethical Committee (Initial protocol N°19/04, decision: 23/02/2004, and successive amendments). Written informed consent was obtained from each participant.

### Assessment of falls

Self-reported fall data was collected prospectively over 12 months after the 2011 assessment (i.e., 6 years after the baseline assessment), using monthly calendars that participants returned by mail via prepaid and preaddressed envelopes, with phone call reminders as currently recommended [24, 25]. A fall was defined as unintentionally coming to rest on the floor or a lower surface, outside sport activity [24]. The circumstances and consequences of the fall, including any consultation triggered by the fall, were also recorded. A fall was further qualified as injurious if the participant reported an injury (fractures and/or soft tissue lesions) that required an outpatient consultation or a hospitalization. Based on these data, previous research, and according to recent falls prevention guidelines [3, 14, 20], participants were categorized into 3 groups: a) non-fallers; b) one-time benign fallers (i.e., reported a single non-injurious fall); c) serious fallers (i.e., reported multiple and/or injurious falls).

Participants who failed to return their diary on time were contacted by phone to get missing information. Participants with incomplete fall diary but who had fallen more than one time or had an injurious fall were included as serious fallers even if they completed less than 11 out of 12 diaries (*n* = 14). Overall, 100 patients (9.9%) were excluded because their fall status remained uncertain, or because they were admitted to a nursing home during the 12 month-period.

### Assessment of cognitive and EF performance

Global and selective cognitive performance was assessed by the following tests:

1. ***Global cognitive function*** was assessed by the Mini-mental State Examination (MMSE), with a score < 24/30 considered as abnormal.
2. ***Processing speed*** was assessed by the trail making test (TMT), part A (TMT-A), consisting in connecting encircled digits from 1 to 25 in numerical order as fast as possible, while recording completion time. Participants in the quintile with worst EF performance (20% with the longest test duration in the sample) were defined as having poor performance.
3. ***Executive functions (EF)*** were assessed with three tests, most frequently used in both clinical routine and research on executive functions [26].

First, TMT, part B (TMT-B) was performed, consisting in connecting circles containing either a number or a letter in alternating sequence as quickly as possible (1-A, 2-B, etc.). Participants in the lowest quintile (20% with the longest test duration in the sample) were considered as having poor performance. The TMT ratio was computed as the time to complete TMT-B minus the time to complete TMT-A divided by the time to complete TMT-A (i.e., TMT-B – TMT-A/TMT-A). By removing the speed element, the ratio better isolates the executive component of the test, with higher ratio indicating poorer EF performance [27]. Participants in the quintile with worst EF performance (i.e., 20% of the sample with the highest ratio) were defined as having poor EF performance.

Second, the Clock Drawing test (CDT) was performed. Participants received a blank sheet of paper and were asked to draw a clock face, to place the hours around the clock and then to draw the hands to indicate ten after eleven. This test explores EF including planning, selective attention, motor sequencing, and monitoring of the task [28, 29]. Scores below 8/10 were considered abnormal [30].

Finally, verbal fluency (VF) was assessed with participants asked to cite as many names of vegetables or fruits as possible in one minute. The final score corresponds to the total number of correct words.

***Decline in EF*** between 2005 and 2011 was defined as follows, according to clinically meaningfull changes: a) CDT: loss of ≥2 points; b) VF: loss of ≥3 points; c) TMT-B: test duration increased ≥10%.

### Covariates

Education was categorized into four groups, from basic school to university level. Conditions associated to fall risk in previous research were selected as covariates. These included neurologic (stroke, TIA, Parkinson’s disease), musculo-skeletal (arthrosis, osteoporosis), as well as other chronic diseases (hypertension, congestive heart failure, other cardiac diseases, chronic pulmonary diseases, diabetes, and obesity). The use of psychoactive drugs for depression, sleep, or anxiety was recorded. Depressive symptoms were assessed using Whooley’s two questions: “During the past month, have you often been bothered by feeling down, depressed or hopeless?” and “During the past month, have you often been bothered by little interest or pleasure in doing things?” [31]. Visual acuity was measured with a Snellen letter test chart. Subjective memory impairment was assessed by asking participants if they had memory complaints that affected their daily life over the last six months.

Walking speed was measured using a stopwatch with the participant walking at self-selected speed over 20 m in a well-lit walkway. A walking aid was allowed if necessary.

Balance was assessed by standing, eyes open, without talking during ten seconds, first on both feet side-by-side. Light balance impairment was defined as unsteadiness while standing but being able to complete the ten second test. Moderate balance impairment was defined as failure or refusal to complete the test.

The level of physical activity was assessed using two questions. Participants were asked, first, how frequently they went outdoors (at least five days a week vs less often) and, second, how much time they spent outdoors (at least 30 min vs less).

Finally, information was collected about self-reported history of falls over the 12-month period before the baseline (2005) and the follow-up (2011) assessments. More precisely, participants had to report whether they fell over year 2004 and year 2010, respectively.

### Statistical analyses

Baseline characteristics of participants, including performance in cognitive tests, were summarized by using simple statistics (mean, percentages). Bivariate analysis to compare participants’ characteristics by 2011 fall status used Chi-square tests, or Fisher’s exact test when a sugroup included less than 10 individuals for categorical variables, and ANOVA for continuous variables.

*Association between baseline EF performance and fall status.* A multivariate multinomial logistic regression model was performed separately for each cognitive test that showed a statistically significant association with fall status in bivariate analyses. Regarding TMT, the group in the lowest quintile, i.e. with the worse performance, was compared to the rest of the sample. Covariates were selected based on their significant association with fall status in bivariate analysis, to estimate adjusted Relative Risk Ratios (adjRRR). The absence of collinearity (defined as a Variable Inflation Factors (VIF) > 10) across the variables entered in the multivariable models was verified.

*Association between EF decline and fall status.* To assess the association between decline in EF performance between 2005 and 2011, and fall status over the 12-month follow-up period (following 2011 assessment), analyses were performed a) using each of the three variables (TMT-B, TMT ratio, verbal fluency) defining decline in EF separately; b) using an overall measure of EF decline defined as decline in any one of the 3 measures.

*Subgroup and supplementary analyses* Bivariate and multivariate analyses investigating the association between EF performance and fall status 6 years later were performed among fallers, using one-time benign fallers as reference group.

Finally, a supplementary analysis was performed to compare the cognitive performance in participants included in the analysis to the cognitive performance of those excluded (*N* = 96) because of incomplete fall diaries, to determine whether these latter had poorer cognitive performance.

### Sensitivity analysis

To overcome potential recall bias in fall reporting caused by cognitive impairment, all analyses were repeated excluding participants with cognitive impairment at the 2011 interview (MMSE < 24 or/and 3-word recall < 3/3), as well as participants with subjective cognitive complaints.

Statistical analyses were performed with Stata, version 14. Statistical significance was set at *p* < 0.05.

## Results

Participants (*n* = 96) excluded because of incomplete falls data had worse performance than participants included in the analysis at the MMSE (score < 24/30: 20.2% vs 5.5%, *p* < 0.001), as well as TMT tests (mean TMT-A: 56.8 ± 22.7 vs 47.7 ± 18.2, *p* < 0.001; mean TMT-B: 134.2 ± 61.5 vs 114.0 ± 49.2, *p* < 0.001). In addition, they were also more likely to report previous recurrent falls at baseline (12.0% vs 4.2% in included participants, *p*.003).

At the end of the 12-month follow-up of incident falls, 605 participants (66.8%) did not report any fall, 118 (13.0%) reported one single non-injurious fall, and 183 (20.2%) reported serious falls.

Characteristics of the entire study population (*n* = 906) and their comparisons by fall status are summarized in Table 1. The prevalence of several characteristics such as depressive symptoms, pain, psychotropic drug use, and history of falls (both in 2004 and 2010) increased steadily across fall groups, from non-fallers to one-time and to serious fallers, whereas gait speed decreased. In contrast, one-time benign fallers tended to have a higher level of education and less subjective memory impairment than non-fallers and serious fallers. Finally, there was no difference accross fall status group in physical activity as measured by the frequency and the duration of outdoor mobility.

**Table 1:** Characteristics of participants at baseline and their comparisons by fall status at 6-year

**Characteristics**	**Total**	**Non-fallers**	**One-time benign fallers**	**Serious fallers**	*P*-value*
	(*N* = 906)	(*N* = 605, 66.8%)	(*N* = 118, 13.0%)	(*N* = 183, 20.2%)	
**Age (years, mean ± SD)**	69.0 ± 1.4	69.0 ± 1.4	69.0 ± 1.3	68.9 ± 1.4	.875
**Women (%)**	59.8	54.2	70.3	71.6	** < .001**
**Education (%)**					
** Basic school**	22.1	23.0	14.4	24.2	**.030**
** Apprenticeship**	40.7	42.7	39.8	34.3	
** High school**	24.7	22.0	34.8	27.0	
** University**	12.6	12.3	11.0	14.6	
**Stroke/TIA (%)**	1.7	1.5	1.7	2.2	.672
**Parkinson (%)**	0.4	0.2	0.9	1.1	.109
**Arthritis (%)**	36.1	33.9	39.0	41.4	.143
**Osteoporosis (%)**	10.2	9.4	7.6	14.4	.107
**Visual impairment (%)**	1.8	1.3	0.9	3.8	.088
**Other chronic diseases (%)**	55.6	56.0	51.7	56.9	.640
**Depressive symptoms (%)**	25.0	22.2	23.7	35.4	**.001**
**Pain (%)**	65.3	61.9	69.5	73.8	**.008**
**Psychotropic drug use (%)**	19.5	16.6	24.1	26.3	**.007**
**Subjective memory impairment (%)**	9.5	8.9	6.9	13.4	.123
**Balance impairment (%)**					
** None**	72.7	74.2	72.0	68.0	.341
** Light**	18.1	17.5	19.5	18.9	
** Moderate**	9.3	8.3	8.5	13.1	
**Walking speed (m/s, mean SD)**	1.17 ± 0.18	1.18 ± 0.18	1.15 ± 0.16	1.13 ± 0.19	**.035**
**Going outdoor frequently (%)**	79.5	79.8	78.8	79.7	.971
**More than 30 min outdoor (%)**	72.5	71.2	78.0	73.1	**.**314
**History of falls (over year 2004 (%)**					
** None**	82.9	87.4	74.6	72.9	** < .001**
** One**	12.9	9.4	20.3	19.9	
** Two or more**	4.2	3.1	5.1	7.2	
**History of falls (over year 2010) (%)**					
** None**	77.1	83.4	73.7	58.2	** < .001**
** One**	17.8	14.1	22.0	27.5	
** Two or more**	4.1	2.5	4.3	14.3	

^*^P-value from Chi-square test or Fisher’s exact test for categorical variables; or ANOVA test for continuous variables

One-time benign fallers were defined as participants who reported a single non-injurious fall

Serious fallers were defined as participants who reported one injurious (i.e., participant reported an injury, a fracture and/or soft tissue lesions requiring an outpatient consultation or a hospitalization) or two or more falls

### Relationship between baseline EF performance and prospective falls

Table 2 provides the results of cognitive tests in the population at baseline and their comparisons across fall groups. At baseline, 5.5% of participants were cognitively impaired by MMSE (score < 24), and 17.9% had abnormal CDT (score ≤ 7). Mean time to complete TMT-A and B were 48 ± 18 s and 114 ± 49 s, respectively. The cut-off value for the worst quintile of TMT-A corresponded to a time of ≥ 59 s, while for TMT-B, the cut-off was ≥ 144 s.

**Table 2:** Performance in cognitive tests at baseline and their comparisons by fall status at 6-year

	**Total**	**Non-fallers**	**One-time benign fallers**	**Serious fallers**	*P*-value*
	(*N* = 906)	(*N* = 605, 66.8%)	(*N* = 118, 13.0%)	(*N* = 183, 20.2%)	
**MMSE score (mean ± SD)**	27.8 ± 1.8	27.8 ± 1.8	28.1 ± 1.6	27.8 ± 1.9	.160
**Abnormal MMSE (%)**	5.5	5.2	3.4	7.9	.232
**Abnormal CDT (%)**	17.9	18.3	15.3	18.0	.728
**Verbal fluency (mean ± SD)**	19.5 ± 4.7	19.2 ± 4.6	20.2 ± 4.6	20.2 ± 4.8	**.023**
**TMT-A (mean ± SD)**	48 ± 18	48 ± 19	46 ± 14	49 ± 19	.804
**TMT-A, Worst quintile (≥ 59 s) (%)**	18.6	19.3	14.4	19.3	.451
**TMT-B (mean ± SD)**	114 ± 49	116 ± 90	105 ± 42	114 ± 51	**.068**
**TMT-B, Worst quintile (≥ 144 s) (%)**	19.8	22.0	9.4	19.3	**.007**
**TMT ratio, Worst quintile (%)**	19.9	22.2	8.6	19.9	**.003**

^*^P-value from Chi-square test for categorical variables and ANOVA for continuous variables

*MMSE*   Mini-mental state evaluation, *CDT*   Clock drawing test, *TMT*   Trail making test

TMT ratio: (TMT-B – TMT-A)/TMT-A

Comparisons of cognitive performance by fall status in bivariate analysis provided heterogeneous results. Whereas results of global cognition (MMSE, CDT) as well as TMT-A did not differ across fall groups, EF performance (TMT-B, TMT-B worst quintile, TMT ratio) appeared best preserved among one-time benign fallers as compared to the other two groups. Verbal fluency was the only EF measure that did not follow this pattern with a significantly worse performance among non-fallers as compared to the two groups of fallers.

In multivariate regression analysis that adjusted for covariates significantly associated to fall status in bivariate analysis (Table 3), participants with worst performance in TMT-B (adjRRR_TMT-B worst quintile_ = 0.38, 95% CI:0.19–0.75, *p* = 0.006) and worse TMT ratio (adjRRR_TMT ratio worst quintile_ = 0.31, 95% CI:0.15–0.64, *p* = 0.001) were significantly less likely to report a single non-injurious fall than no fall at all. In contrast, participants with worse EF performance did not have an increased risk of serious falls.

**Table 3:** Results of multivariable multinomial regression investigating the association between executive function at baseline and falls status at 6-years

	**Non-fallers**	**One-time benign fallers**			**Serious fallers**		
	(*N* = 605)	(*N* = 118)			(*N* = 183)		
		RRR	95% CI	*P*-value	RRR	95% CI	*P*-value
**Verbal fluency score**	Ref	1.01	0.97–1.06	.609	1.02	0.98–1.07	.295
**TMT-B, worst quintile (≥ 59 s)**	Ref	0.38	0.19–0.75	**.006**	0.87	0.54–1.41	.575
**TMT ratio, worst quintile (≥ 144 s)**	Ref	0.31	0.15–0.64	**.001**	0.94	0.59–1.48	.791

Ref.: reference category

Model was adjusted for sex, education, pain, psychotropic drug use, depressive symptoms, walking speed, fall in the previous year, as these variables were significantly associated with the outcome in bivariate analyses

*TMT*   Trail making test, *TMT-B * Trail making test, part B

In the three above-mentioned models that tested EF performance (i.e., verbal fluency, TMT-B, TMT ratio), the only variables that remained significantly associated with reporting falls were female gender and reporting a fall in the previous 12-month period at baseline assessment (Supplementary Tables).

### Relationship between decline in EF performance and prospective falls

Between 2005 and 2011, 72.7% of the sample declined in at least one of the three EF tests: 19.8% declined in the CDT test, 37.3% declined in VF test and 46.2% declined in TMT-B (Table 4). The proportion of participants with EF decline did not differ across fall groups, even though this proportion tended to be higher in one-time benign fallers. The sensitivity analysis using a decline in any EF test as independent variable provided similar results.

**Table 4:** Proportion of participants with a decline in each specific test of executive function (EF) between 2005 and 2011, and their comparisons by fall status at 6-years

	**Total**	**Non-fallers**	**One-time benign fallers**	**Serious fallers**	*P*-value*
	(*N* = 906)	(*N* = 605)	(*N* = 118)	(*N* = 183)	
**Decline in CDT**	19.8	18.2	25.4	21.5	.160
**Decline in Verbal fluency**	37.3	35.1	39.8	42.6	.158
**Decline in TMT-B**	46.2	45.6	50.0	45.3	.686
**Decline in any EF test**	72.7	71.6	75.7	74.6	.555

^*^P-value from Chi-square test

CDT = clock drawing test, TMT = trail making test

Decline in CDT defined as a loss ≥ 2 points between 2005 and 2011

Decline in Verbal fluency defined as a loss ≥ 3 points between 2005 and 2011

Decline in TMT-B defined as a ≥ 10% increase in the time to complete TMT-B between 2005 and 2011

### Subgroup and sensitivity analyses

In multivariate logistic regression analysis restricted to fallers, participants with worse EF performance in TMT-B (adjOR_TMT-Bworst quintile_ 1.86, CI 95% 0.98–3.53, *p* = 0.059) and worse TMT ratio (adjOR _TMT ratio worst quintile_ 1.84, CI 95% 0.98–3.43, *p* = 0.057) had increased odds of reporting serious falls, but these associations did not achieve statistical significance (Table 5).

**Table 5:** Results of the subgroup analysis among fallers: multivariate logistic regression analysis investigating the association between executive function at baseline and fall status at 6-years

	**One-time** **benign fallers** **(*n* = 118)**	**Serious fallers (*n* = 183)**		
		**OR**	**(95% CI)**	***P*-value**
**TMT-B,** **worst quintile** **(≥ 144 s)**	Ref	1.86	(0.98–3.53)	.059
**TMT ratio,** **worst quintile**	Ref	1.84	(0.98–3.43)	.057

Ref.: reference category; 95% CI:95% confidence interval

TMT = trail making test; TMT-B: trail making test, part B

TMT ratio: (TMT-B – TMT-A)/TMT-A

Adjusted for depressive symptoms (only variable that remained significantly associated)

Additional sensitivity analyses were also performed to further investigate whether a potential bias in fall recall due to memory impairment could modify the association between EF performance and fall status. Results were unchanged in two separate analyses that excluded a) participants with cognitive impairment (MMSE < 24 or/and < 3/3 at word recall test); b) participants with subjective cognitive complaints (data not shown).

## Discussion

This study examined whether EF performance in community-dwelling older adults was associated with falls occurrence six years later, and whether this association differed according to the number and severity of falls.

Contrary to our hypothesis, participants with worse EF performance at baseline were actually significantly less likely to report a single non-injurious fall than no fall. In contrast, in a subgroup analysis restricted to fallers, poor EF performance tended to be associated with higher odds of serious falls (i.e., repeated or injurious falls). Most likely, these results are explained by the very similar EF performance in non-fallers and serious fallers as compared to one-time benign fallers. Indeed, except for the verbal fluency test, one-time non-injurious fallers performed systematically and sometimes (i.e., TMT-B and TMT-ratio) significantly better than the other two groups. A misclassification in falling groups appears unlikely as most other well-known risk factors for falls [6, 32, 33] such as walking speed, psychotropic drug use, depressive symptoms, previous stroke, and previous falls, showed incremental prevalence from non-fallers, to one-time benign fallers, and to serious fallers. Indeed, all observed associations remained in multivariate analyses that adjusted for these potential confounders, as well as for education, another characteristic that differed significantly between one-time benign fallers (more educated) and the two other groups. Likewise, activity and mobility levels did not differ across falling groups to suggest significant differences in exposition to falls. Finally, results of sensitivity analyses excluding participants with objective and subjective memory problems were unchanged, further excluding a potential selective bias in fall recall among non-fallers.

These results differ from those of Mirelman A et al. [7] who observed that an index of EF measured at baseline predicted falls over a five-year follow-up period. This likely resulted from differences in participants’ characteristics, those in the present study being younger (69.0 ± 1.4 vs 76.4 ± 4.5 years), and somewhat less likely to report at least one fall in the previous year (17.1% vs 23%) even though they had slower walking speed (1.17 ± 0.18 vs 1.23 ± 0.22 m/s). Unfortunately, it is not possible to compare EF performance as a specific index was used in Mirelman’s study. Another study, that defined falls only as those leading to a hospitalization or outpatient care, found an association with the delta TMT (difference in TMT-B minus TMT-A), but not with TMT-B at 5-year follow-up. Interestingly, mean age in this cohort (72.0 ± 9.8 years) was closer to ours, but reported mean TMT-B time is quite unusually short (36.7 ± 22.4 and 28.5 ± 16.4 in fallers and non-fallers, respectively, vs 114 ± 49 in the present study). Our sample seems nevertheless rather representative of the general population of their age as the median TMT-B of 100 s is close to the normative value of 97 s found in a similar age group (70 -74 years) [34]. 

An alternative explanation might be that EF tests used in the present study were too general measures of EF and lacked sensitivity as compared to computerized test battery as used in some studies that reported significant association between EF and falls [2, 7]. In particular, these tests that were selected based on their extensive use in clinical routine and in research do not assess well inhibitory control, an EF function that has been specifically associated with falls in people with cognitive impairment [2].

Overall, results from the present study could suggest that EF performance might be a weaker predictor in a population of relatively young older adults, as suggested also by the limited amount of variance (about 5%) in falls status explained by the different models in our analyses. However, results from the subgroup analysis among fallers still showed a trend toward increased odds of serious falls among those with worse EF, consistent with a meta-analysis that found twice the risk of injuries in older persons with impaired EF [3]. Indeed, these results extend previous knowledge in showing that this association, already described in several cohorts of older persons [35, 36], is also observed in younger elderly persons. The profile of participants who reported no falls, with cognitive and EF characteristics closer to those of serious fallers, likely explain some of these differences. These results may also suggest that, in this relatively young cohort of older adults, impaired EF mainly translates into a higher risk of serious falls because of increased risk-taking behavior, inappropriate evaluation of the environment, and/or impaired protective reactions.

Another original contribution of the present work was to examine the dynamic association between a decline in EF performance over 6 years and prospective falls. Again, no significant association was observed. Although this results could reflect the absence of a true association, several alternative explanations can be proposed. First, the EF tests used could lack sensitivity to change. This seems however unlikely as almost three quarters of participants showed some decline as defined in this study. Indeed, an alternative explanation could be that our definition of decline was too loose and lacked specificity. Finally, these negative results might stem from differences in study population, and caution should therefore be applied when interpreting these results.

Strengths of this study include its relatively large study sample, the long follow-up, and the prospective collection of information regarding falls, using monthly calendars and phone calls [37]. These reference methods for fall ascertainment provided detailed information about falls, thus allowing to identifying recurrent and injurious falls. An additional strength is the use of several measures of EF, including TMT ratio that allow to control for psychomotor speed and better isolate the cognitive flexibility component of the test.

A limitation of this study is the exclusion of participants with incomplete falls data (*n* = 96) who had significantly poorer baseline cognitive performance and more likely reported previous recurrent falls. Thus, these participants would have been quite likely to fall again and be classified in one the one-time or multiple fallers group than in non-fallers. Although they represent only 10% of the initial study sample, exclusion of these participants not only limited the study’s power to detect an association between EF and falls, but also potentially lead to an underestimation of this association.

In conclusion, participants with worse EF performance at baseline were less likely to report a single non-injurious fall than no fall over the 12-month fall recording period. No association was either observed between EF decline and falls status 6 years later. However, among fallers, those with worse EF tended to have higher odds of reporting serious falls (i.e., repeated or injurious falls). Future studies should further investigate whether, in active and relatively young older adults, slight EF impairment mainly translates into higher risk of serious falls among fallers because of increased risk-taking behavior, inappropriate evaluation of the environment, and/or impaired protective reactions.

## Supplementary Information

**Additional file 1: Fig. 1.** Flow diagram of Lc65+ participants in this study from 2005 to 2011. **Table 1.** Results of multivariable multinomial regression investigating the association between verbal fluency in 2005 and falls status in 2011. **Table 2.** Results of multivariable multinomial regression investigating the association between TMT-B performance in 2005 and falls status in 2011. **Table 3.** Results of multivariable multinomial regression investigating the association between TMT ratio in 2005 and falls status in 2011.

## Data Availability

Are not publicly available, but might be requested upon first author.
