# Supplementary material for: Executive function and prospective falls: a 6-year longitudinal study in community-dwelling older adults
Source: BMC Geriatr. 2023 Mar 10;23:140. doi: 10.1186/s12877-023-03790-9 (PMC10007810; doi:10.1186/s12877-023-03790-9)
Supplement: Supplementary file 1 — Additional file 1: Fig. 1. Flow diagram of Lc65+ participants in this study from 2005 to 2011. Table 1. Results of multivariable multinomial regression investigating the association between verbal fluency in 2005 and falls status in 2011. Table 2. Results of multivariable multinomial regression investigating the association between TMT-B performance in 2005 and falls status in 2011. Table 3. Results of multivariable multinomial regression investigating the association between TMT ratio in 2005 and falls status in 2011. [file 12877_2023_3790_MOESM1_ESM.docx]

**Supplementary material**

**Baseline sample in 2005**

(N=1'422)

**Lost to follow-up (**N=255)

- Died : n=156
- No answer : n=99

**Eligible population in 2011**

(N=1'167)

**Incomplete participation (**N=165)

- Questionnaire only : n=150
- Proxy : n=11

**Participated in 2011**

**data collection**

(N=1’006)

**Incomplete fall information** (N=96)

Nursing home admission (N=4)

**Final study sample**

(N=906)

**Figure 1**: Flow diagram of Lc65+ participants in this study from 2005 to 2011

**Table 1.** Results of multivariable multinomial regression investigating the association between verbal fluency in 2005 and falls status in 2011

|  | Non-fallers | One-time benign fallers | | | Serious fallers | | |
| --- | --- | --- | --- | --- | --- | --- | --- |
|  | (N=605) | (N=118) | | | (N=183) | | |
|  |  | RRR | 95% CI | P-value | RRR | 95% CI | P-value |
| Verbal fluency score | Ref. | 1.01 | 0.97-1.06 | .609 | 1.02 | 0.98-1.07 | .295 |
| Women | Ref. | 1.76 | 1.08-2.86 | .024 | 1.55 | 1.00-2.40 | .048 |
| Education (ref : basic school):  Apprenticeship  High school  University | Ref. | 1.71  2.76  1.86 | 0.92-3.20  1.43-5.30  0.83-4.24 | .091  .002  .140 | 0.79  1.21  1.36 | 0.48-1.29  0.71-2.05  0.72-2.56 | .342  .487  .340 |
| Pain | Ref. | 1.24 | 0.79-1.95 | .339 | 1.23 | 0.82-1.85 | .321 |
| Use of psychotropes | Ref. | 1.56 | 0.92-2.63 | .099 | 1.20 | 0.74-1.93 | .460 |
| Mood disorders | Ref. | 0.90 | 0.54-1.49 | .675 | 1.44 | 0.94-2.20 | .093 |
| Gait speed (m/s) | Ref. | 0.67 | 0.19-2.30 | .524 | 0.73 | 0.25-2.13 | .569 |
| History of falls (over year 2004)  One  Two or more | Ref. | 1.60  1.57 | 0.95-2.67  0.54-4.58 | .075  .409 | 2.23  6.92 | 1.43-3.48  3.40-14.1 | .000  .000 |

**Table 2.** Results of multivariable multinomial regression investigating the association between TMT-B performance in 2005 and falls status in 2011

|  | Non-fallers | One-time benign fallers | | | Serious fallers | | |
| --- | --- | --- | --- | --- | --- | --- | --- |
|  | (N=605) | (N=118) | | | (N=183) | | |
|  |  | RRR | 95% CI | P-value | RRR | 95% CI | P-value |
| TMT-B, worst quintile | Ref. | 0.38 | 0.19-0.75 | .006 | 0.87 | 0.54-1.41 | .575 |
| Women | Ref. | 1.89 | 1.18-3.02 | .008 | 1.74 | 1.14-2.65 | .010 |
| Education (ref : basic school):  Apprenticeship  High school  University | Ref. | 1.66  2.53  1.64 | 0.87-3.17  1.30-4.94  0.71-3.81 | .121  .006  .247 | 0.79  1.25  1.39 | 0.48-1.30  0.73-2.13  0.73-2.66 | .347  .442  .316 |
| Pain | Ref. | 1.29 | 0.82-2.03 | .270 | 1.18 | 0.78-1.77 | .437 |
| Use of psychotropes | Ref. | 1.60 | 0.94-2.71 | .081 | 1.22 | 0.75-1.97 | .427 |
| Mood disorders | Ref. | 0.96 | 0.57-1.60 | .874 | 1.48 | 0.96-2.27 | .074 |
| Gait speed (m/s) | Ref. | 0.72 | 0.21-2.48 | .607 | 0.83 | 0.28-2.44 | .739 |
| History of falls (over year 2004):  One  Two or more | Ref. | 1.62  1.56 | 0.96-2.71  0.53-4.58 | .070  .423 | 2.22  7.15 | 1.42-3.48  3.43-14.91 | .000  .000 |

**Table 3.** Results of multivariable multinomial regression investigating the association between TMT ratio in 2005 and falls status in 2011

|  | Non-fallers | One-time benign fallers | | | Serious fallers | | |
| --- | --- | --- | --- | --- | --- | --- | --- |
|  | (N=605) | (N=118) | | | (N=183) | | |
|  |  | RRR | 95% CI | P-value | RRR | 95% CI | P-value |
| TMT ratio, worst quintile | Ref. | 0.31 | 0.15-0.64 | .001 | 0.94 | 0.59-1.49 | .791 |
| Women | Ref. | 1.92 | 1.20-3.08 | .006 | 1.74 | 1.14-2.65 | .010 |
| Education (ref : basic school):  Apprenticeship  High school  University | Ref. | 1.79  2.79  1.82 | 0.94-3.41  1.43-5.42  0.78-4.20 | .075  .003  .164 | 0.80  1.27  1.44 | 0.49-1.32  0.75-2.16  0.76-2.72 | .380  .377  .266 |
| Pain | Ref. | 1.32 | 0.84-2.08 | .235 | 1.17 | 0.78-1.76 | .450 |
| Use of psychotropes | Ref. | 1.62 | 0.96-2.75 | .072 | 1.22 | 0.75-1.97 | .426 |
| Mood disorders | Ref. | 0.89 | 0.53-1.49 | .663 | 1.46 | 0.95-2.25 | .082 |
| Gait speed (m/s) | Ref. | 0.87 | 0.25-3.02 | .824 | 0.86 | 0.29-2.53 | .787 |
| History of falls (over year 2004):  One  Two or more | Ref. | 1.64  1.63 | 0.97-2.75  0.55-4.83 | .062  .383 | 2.22  7.29 | 1.42-3.47  3.50-15.21 | .000  .000 |
